# Supplementary material for: Effects of Halogen Lamp and Traditional Sun Drying on the Volatile Compounds, Color Parameters, and Gel Texture of Gongliao Gelidium Seaweed
Source: Foods. 2023 Dec 17;12(24):4508. doi: 10.3390/foods12244508 (PMC10743001; doi:10.3390/foods12244508)
Supplement: Supplementary file 1 [file foods-12-04508-s001.zip › foods-2715144-supplementary.pdf]

Supplementary Table S1. Correlation coefficients of color, texture, and sensory evaluation results of *Gelidium* seaweed

	Appearance	Color	Texture	Flavor	Overall	Fishy odor	L^*	a^*	b^*	Hardness	Springiness
Appearance	1										
Color	0.972^{**}	1									
Texture	0.882^{*}	0.770	1								
Flavor	0.949^{**}	0.856^{*}	0.980^{**}	1							
Overall	0.933^{**}	0.846^{*}	0.989^{**}	0.992^{**}	1						
Fishy odor	-0.953^{**}	-0.870^{*}	-0.923^{**}	-0.977^{**}	-0.959^{**}	1					
L^*	0.929^{**}	0.899^{*}	0.755	0.858^{*}	0.804	-0.878^{*}	1				
a^*	-0.045	0.043	-0.056	-0.122	-0.057	0.217	-0.290	1			
b^*	0.958^{**}	0.937^{**}	0.795	0.892^{*}	0.858^{*}	-0.928^{**}	0.971^{**}	-0.297	1		
Hardness	0.945^{**}	0.939^{**}	0.801	0.878^{*}	0.858^{*}	-0.884^{*}	0.945^{**}	-0.247	0.981^{**}	1	
Springiness	0.895*	0.802	0.883^{*}	0.931^{**}	0.894^{*}	-0.918^{**}	0.935^{**}	-0.398	0.931^{**}	0.922^{**}	1

*indicate significance at $p < 0.05$

** indicate significance at $p < 0.01$

Supplementary Table S2. Volatile compounds of Fresh and untreated *Gelidium* seaweed

	Compound	RI	Concentration* (ng/ml)
1	acetic acid	600	46.9
2	hexanal	801	281
3	2-hexenal	844	61.3
4	heptanal	895	499
5	1-octen-3-ol	972	97.4
6	octanal	998	235
7	3,5-octadien-2-ol	1037	62.3
8	2,6-nonadienal	1148	43.3
9	β-cyclocitral	1213	46.2
10	2-decenal	1241	28.5
11	α-ionone	1412	254
12	β-ionone	1471	379

*Semi-quantitated

Supplementary Table S3. Volatile compounds of dried *Gelidium* seaweed (three washing and sun drying cycle)

	Compound	RI	Concentration* (ng/ml)
1	hexanal	824	178
2	heptanal	899	92.3
3	2-heptenal	950	336
4	octanal	1001	295
5	2,4-heptadienal	1010	200
6	3-ccten-2-one	1040	250
7	2-octenal	1061	307
8	1-octanol	1073	191
9	3,5-octadien-2-one	1089	94.4
10	nonanal	1103	332
11	2,6-nonadienal	1151	28.4
12	2-nonenal	1160	98.3
13	2,4-nonadienal	1192	185
14	β-cyclocitral	1217	25.0
15	2-decenal	1245	206
16	2,4-decadienal	1290	179
17	2-undecenal	1359	64.3
18	dodecanal	1397	25.0
19	α-ionone	1412	63.5
20	β-ionone	1470	22.4
21	epoxy-β-ionone	1479	28.1
22	1-pentadecene	1484	21.9
23	tridecanal	1498	11.3
24	octadecenal	1559	25.2
25	tetradecanal	1599	27.6

*Semi-quantitated

Supplementary Table S4. Volatile compounds of dried *Gelidium* seaweed (seven washing and drying cycles)

	Compound	RI	Concentration* (ng/ml)
1	hexanal	801	138
2	2-hexenal	844	29.6
3	heptanal	895	22.3
4	2-heptenal	950	177
5	octanal	998	287
6	3-octen-2-one	1033	55.3
7	2-octenal	1057	61.6
8	1-octanol	1070	49.5
9	nonanone	1087	8.90
10	nonanal	1100	252
11	2-nonenal	1142	137
12	nonadienal	1188	108
13	decanal	1198	46.3
14	β-cyclocitral	1213	15.4
15	2-decenal	1241	645
16	2,4-decadienal	1286	150
17	undecanal	1298	56.0
18	2-undecenal	1354	103
19	undecanol	1363	32.3
20	dodecanal	1397	61.7
21	dodecenal	1456	69.0
22	octadecenal	1462	28.2
23	epoxy-β-ionone	1473	10.1
24	pentadecene	1481	68.7
25	tridecanal	1498	54.0
26	octadecenal	1559	25.7
27	tetradecanal	1599	12.1

*Semi-quantitated.

Supplementary Table S5. Volatile compounds of *Gelidium* seaweed (seven washing and halogen lamp drying cycles)

	Compound	RI	Concentration* (ng/ml)
1	hexanal	801	233
2	2-hexenal	844	31.8
3	2,4-hexadien-1-ol	860	7.45
4	2-heptanone	881	12.1
5	heptanal	895	35.6
6	2-heptenal	945	281
7	1-octen-3-one	969	44.2
8	1-octen-3-ol	972	29.9
9	2,4-heptadienal	990	184
10	octanal	998	169
11	3-octen-2-one	1033	315
12	2-octenal	1057	235
13	3,5-octadien-2-one	1067	44.8
14	1-octanol	1070	143
15	3,5-octadien-2-one	1086	53.7
16	nonanal	1099	292
17	2-nonenal	1142	77.8
18	2,6-nonadienal	1148	14.3
19	decanal	1198	20.8
20	2,4-nonadienal	1188	374
21	β-cyclocitral	1213	18.2
22	2-decenal	1241	112
23	2,4-decadienal	1286	284
24	undecanal	1298	6.77
25	dodecanal	1397	23.3
26	α-ionone	1412	35.7
27	β-ionone	1413	14.4
28	epoxy-β-ionone	1473	18.2
29	tridecanal	1498	34.3

*Semi-quantited.

Supplementary Table S6. Volatile compounds of *Gelidium* seaweed (nine washing and halogen lamp drying cycles)

	Compound	RI	Concentration* (ng/ml)
1	hexanal	796	156
2	2-hexenal	844	26.7
3	2-heptanone	881	5.41
4	heptanal	895	22.3
5	2-heptenal	945	246
6	1-octen-3-one	969	17.4
7	1-octen-3-ol	972	13.1
8	octanal	998	138
9	2,4-heptadienal	1008	112
10	3-octen-2-one	1033	147
11	3,5-octadien-2-one	1036	8.98
12	2-octenal	1057	179
13	1-octanol	1070	45.4
14	3,5-octadien-2-one	1089	20.7
15	nonanal	1098	286
16	2-nonenal	1142	161
17	2,6-nonadienal	1147	4.76
18	2,4-nonadienal	1188	302
19	decanal	1198	37.8
20	β-cyclocitral	1212	8.10
21	2-decenal	1241	474
22	2,4-decadienal	1286	184
23	undecanal	1298	21.2
24	2-undecenal	1354	21.3
25	undecanal	1397	36.6
26	α-ionone	1412	11.9
27	1-pentadecene	1480	36.9
28	tridecanal	1498	36.6

*Semi-quantitated.

Supplementary Table S7. Volatile compounds of *Gelidium* seaweed (twelve washing and halogen lamp drying cycles)

	Compound	RI	Concentration* (ng/ml)
1	hexanal	801	142
2	2-hexenal	844	22.3
3	heptanal	895	15.0
4	1-ccten-3-one	969	7.36
5	octanal	998	139
6	3-octen-2-one	1033	99.5
7	2-octenal	1057	110
8	1-octanol	1070	14.8
9	nonanal	1099	305
10	2-nonenal	1142	214
11	decanal	1198	59.5
12	2,4-nonadienal	1188	264
13	β-cyclocitral	1213	7.43
14	2-decenal	1241	526
15	2,4-decadienal	1286	126
16	undecanal	1298	47.6
17	2-undecenal	1354	95.5
18	dodecanal	1397	43.2
19	α-ionone	1412	12.2
20	2-dodecenal	1456	60.4
21	1-pentadecene	1480	64.1
22	tridecanal	1498	42.2

*Semi-quantitated.
